# Probing Selective Adsorption in Cationic-Polymer Induced Aggregation of Binary Anionic Particulate Dispersions Using Solvent Relaxation NMR

**DOI:** 10.3390/polym14091875

**Published:** 2022-05-04

**Authors:** Wasiu Abdullahi, Martin Crossman, Peter Charles Griffiths

**Affiliations:** 1 School of Science, Faculty of Engineering and Science, University of Greenwich, Chatham Maritime, Kent ME4 4TB, UK; w.o.abdullahi@greenwich.ac.uk; 2 Unilever Research, Port Sunlight, Quarry Road East, Bebington, Wirral CH63 3JW, UK; martin.crossman@unilever.com

**Keywords:** preferential adsorption, phase separation, NMR solvent relaxation

## Abstract

NMR solvent relaxation has been used to characterize the surfaces present in binary anionic particle dispersions, before and after exposure to a cationic polymer. In the polymer-free case, it is shown that the measured specific relaxation rate of the solvent is a population-weighted average of all surfaces present, enabling preferential adsorption to be explored. The addition of the oppositely charged polymer led to phase separation, which was accelerated by gentle centrifugation. The measured relaxation rates and the equilibrium particle concentrations indicate that the cationic hydroxyethylcellulose polymer (HEC LR) exhibited no significant preference for either latex or laponite in binary blends with silica, but a strong preference for TiO_2_. This study illustrates the versatility of solvent relaxation to probe surface area, surface type and dispersion composition in complex formulations.

## 1. Introduction

The preferential interaction of polymers with the different surfaces (or interfaces) present in multi-particle, complex formulations often define the fate of the product during storage, transportation, handling, and application. Mixed particle formulations are common: dispersions such as paints contain lattices, whiteners and pigments [1], sunscreens incorporate TiO_2_ and ZnO as UV filters, [2,3,4] and some cosmetics have been reported to use up to four particle types, including TiO_2_, ZnO, silica and carbon black [5]. These formulations also contain polymers to provide particle stabilization and rheology characteristics.

The interaction of polymers with a particle type in a mixture of particles can vary significantly in the presence of other potential surface adsorbents. Although polymer adsorption is often irreversible, changes in bulk conditions, polymer polydispersity and the presence of other adsorbents can cause polymer desorption. In a multi-particle system where the polymer shows a surface preference, this can influence the adsorbed amount and affect the equilibration time. Ultimately, in a formulated product, this can lead to order-dependent product stability—the equilibration time being controlled by the time in which the preferred particle is added.

Polymers intended to condition a surface during use may encounter extraneous (e.g., dirt) particles; if the polymer shows a greater preference for the extraneous particle, it is washed off along with the dirt particles robbing the target surface of the intended conditioning. This may affect the performance of a product in a varied and uncontrolled manner i.e., when used at different times by the same user, as well as a dependence on the initial cleanliness of the substrate.

Solvent relaxation [6,7,8] has been shown to be a viable and unique technique for quantifying surface structures in complex formulated particle-containing systems—whether those formulations contain surfaces that are “bare” [9] or comprise polymer-coated surfaces [6], or, indeed, polymer-surfactant-surface borne structures [10,11] quantifying directly, and only, the near-surface structures. The technique is not sensitive to any polymer or polymer-surfactant complex in the continuous phase, and, crucially, is not limited by the concentration or opacity of the formulation.

This study investigates whether solvent relaxation is a viable methodology to explore any surface preference of a cationic-modified hydroxyethylcellulose polymer in binary particle mixtures of silica/latex, silica/TiO_2_ and silica/laponite. Silica was used as a model hydrophilic surface [12], latex serves as a model for a hydrophobic surface (e.g., dirt particles) and laponite (clay) serves as a model for a common water contaminant

## 2. Materials and Methods

### 2.1. Materials

**Figure 1:**
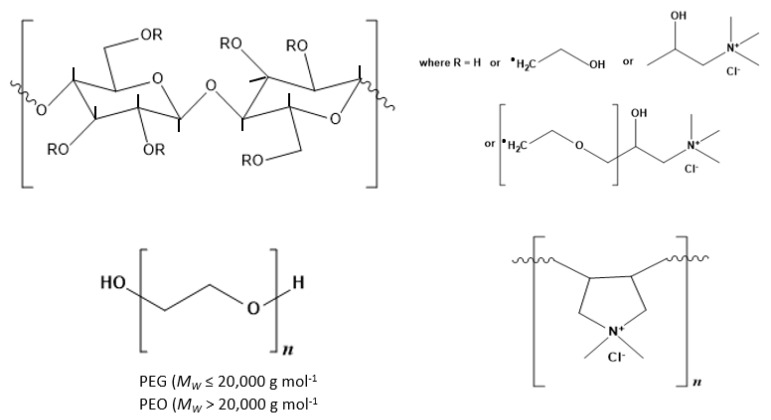
Structure of the polymers studied: hydroxyethylcellulose (HEC) and cationic HEC (cat-HEC) polymers. In HEC, R = H or –(CH2CH2O)_n_H while for cat-HEC, R = H, –(CH_2_CH_2_O)_n_H, or –(CH_2_CH_2_O)_n_CH_2_(CH_2_OH)CH_2_N^+^(CH_3_)_3_Cl−.

Hydroxyethylcellulose (HEC) is a partially substituted hydroxyethyl ether of cellulose. Each hydroxyethyl group can be further derivatized by reacting with ethylene oxide to form a side chain that may be subsequently quaternized i.e., ammonium salts of trimethylammonium derivatives of HEC (Figure 1), to yield cationic polymers whose charge is defined by the extent of charge modification through the degree of substitution expressed as 0.9%. Classified as polyquaternium-10, according to the International Nomenclature of Cosmetic Ingredients (INCI), this family of polymers is manufactured under the trade name UCARE (Dow Chemical Company, Midland, MI, USA). All work here focuses on polymer LR, which was supplied in powder form, and with an average molecular weight estimated to be 500,000 g mol^−1^. Recent SANS analysis [13] of the polymer indicated it exhibited a rodlike solution conformation with each unit within the rod exhibiting a dimension R = 0.8 nm and L = 1 nm i.e., volume = 2–3 nm^3^ whilst analysis of its self-diffusion behavior14 indicates an effective hydrodynamic (spherical) radius of roughly 25 nm [14].

Ludox TM-50 (Sigma-Aldrich, St. Louis, MO, USA), titanium (IV) oxide, anatase (TiO_2_) (Sigma-Aldrich, St. Louis, MO, USA), Laponite-RD (Laponite) (BYK Additives and Instruments, Wesel, Germany) and styrene/acrylic latex dispersion [15], (AkzoNobel Slough, UK) had the characteristics presented in Table 1. The particle size and ζ-potential were determined with a Malvern Zetasizer Nano ZS (Malvern, UK) whilst BET surface area was extracted from analysis by Micromeritics Gemini V 2380 surface area analyser (Norcross, GA, USA).

The water used for all sample preparations had a minimum resistivity of 18.2 MΩ/cm and was obtained from a Purite Select Fusion 80 water purification system. 

The silica and latex dispersions were extensively dialysed against water. The end of the dialysis period was determined by the observation of a constant conductivity of the medium. All dispersions were analyzed gravimetrically to determine the solids concentration.

### 2.2. Methods

Solvent relaxation characterization of particulate dispersions is reliant on just two facets of the system—that the majority of the NMR signal arises from the solvent component and that the surface has an effect of sufficient magnitude on the dynamics of the solvent. These conditions are met by all aqueous dispersions studied to date [16,17], and the majority of non-aqueous ones [18]. Briefly, when a water (or more generally, a solvent) molecule interacts with a surface, its mobility is constrained and the dynamics become anisotropic or restricted. Under these conditions, the efficiency of dipolar relaxation is increased, and the (spin-spin) relaxation time *T*_2_ is shortened; this is termed an *enhancement* in the relaxation **rate**. This is equivalent to observations in high-resolution NMR spectroscopy where peaks become broadened for larger molecules. The *T*_2_ and width of NMR peaks are simply related. In the case of solvent relaxation, one focuses on just the behavior of the solvent, and the peak broadening or shortening of the *T*_2_ is more pronounced when there is a greater amount of surface present as the solvent molecules spend a greater fraction of time *p*_b_ bound at the surface;

(1) $$\frac{1}{T_{2}}=\frac{\left(1-p_{b}\right)}{T_{2f}}+\frac{p_{b}}{T_{2b}}$$

where the relaxation time is converted into a rate $\frac{1}{T_{2}}=R_{2}$ and normalized by the relaxation rate of the water employed to prepare the dispersion, *R*_2,0_, to yield the specific relaxation rate *R_2sp_*

(2) $$R_{2sp}=\frac{R_{2}}{R_{2,0}}-1$$

*R_2sp_*, therefore, scales linearly with the fraction of time (*p*_b_) each water molecule spends at the surface which is assumed to be equivalent to the amount of surface area (*σ*) present, and tends to zero in the absence of (any) surface. The normalization using the water used to prepare the dispersion also has the benefit of reducing the impact of certain artifacts introducing characteristics, such as ionic strength, presence of ions etc. Equation (1) has been shown to have validity over all particle types and concentrations studied to date. 

One might therefore, generalize for a dispersion containing more than a single particle type i.e., *nsurf* discrete particle *surfaces*;

(3) $$\langle R_{2sp}\rangle =\overset{N}{\underset{nsurf=1}{\sum }}R_{2sp}^{enh}\;\sigma$$

where $R_{2sp}^{enh}$ is the enhancement in relaxation rate induced by the specific particle (normalized surface area or concentration if *σ* is recast in that manner) or for the simple binary case;

(4) $$\langle R_{2sp}\rangle =R_{2sp}^{enh\;\left(silica\right)}\sigma_{silica}+R_{2sp}^{enh\;\left(secondary\right)}\sigma_{secondary}$$

This is a key hypothesis that will be tested in the ensuing sections.

The relaxation rates have been determined using a Xigo Nanotools Acorn Arealow field (13 MHz) spectrometer employing the standard Carr–Purcell–Meiboom–Gill (CPMG) pulse sequence. The temperature of the spectrometer and sample stage is regulated to 25 ± 0.5 °C. The instrument macro automatically setup the resonance field position of the spectrometer, optimized the experimental parameters and determined the measured *T*_2_ relaxation time, in triplicate, of the water for 0.5 mL of each sample. Typically, 718 data points (echo cycles) were collected for each scan, with each sample being characterized over four scans with an interpulse spacing (τ of 0.50 ms between the 90° and 180° pulses. A recycle delay of about 5× *T*_1_ was allowed between each cycle to allow full recovery of the magnetization between acquisitions. The Acorn AreaQuant software was used to fit all the relaxation decay curves measured to a single exponential decay using the equation:

(5) My(t)=My(0)exp(−τT2 )

## 3. Results

Here, binary particle dispersions have been studied by solvent relaxation NMR, with the view of assessing whether changes in the average (or measured) solvent relaxation time (or rate) offer a mechanism to determine the surface area of each particle type present in such binary particle dispersions, and, thereafter, through changes in that experimentally determined surface area, any preferential adsorption of a polymer to one of the two surfaces. The systems have been selected to have different particle sizes/surface areas and relaxation rate enhancements. The effect of a given surface area on the measured property $R_{2sp}^{enh\;\left(silica\right)}=0.24\;mL\;g^{-1}$, $R_{2sp}^{enh\;\left(latex\right)}=0.48\;mL\;g^{-1}$, $R_{2sp}^{enh\;\left(laponite\right)}=4.0\;mL\;g^{-1}$ and $R_{2sp}^{enh\;\left(TiO2\right)}=14\;mL\;g^{-1}$. The polymer and particles bear opposite charges, so changes in the surface area are expected to be induced through macroscopic phase separation, as per our previous study [19] i.e., polymer-coated particles aggregate leaving a coexisting polymer-free bare particle dispersion. It is worth highlighting at this stage, that all the particles bear an anionic charge, and have comparable ζ-potentials.

### 3.1. Simple Particle Dispersions

Three different binary particle mixtures have been investigated, each mixture containing silica (Ludox). Two experimental protocols were explored: (1) varying the total particle surface area with a constant ratio of the two particles ($\frac{\sigma_{silica}}{\sigma_{secondary}}$) i.e., f_n_ ($\sigma_{silica}+\sigma_{secondary})$; or (2) varying the total particle surface area in the presence of a fixed amount of one of the two particles i.e., f_n_ ($\sigma_{silica})+\sigma_{secondary}$. The solvent relaxation data are presented in compound Figure 1, for Ludox/latex (top row), Ludox/laponite (middle 2 rows) and Ludox/TiO_2_ (bottom row), in terms of particle concentrations (left column) and surface areas (right column).

In all cases, the solvent relaxation rate shows a linear dependence on particle concentration (and *inter alia* surface area), with that value also being commensurate with the nature of the particles present, i.e., Equation (4) is valid. This is manifested through the effective slopes of the relaxation rate *vs.* particle surface area behaviors being composition weighted averages, i.e., lying between the two limiting cases (protocol 1) or being laterally and vertically offset in the case of the fixed concentration particle experiment (protocol 2).

**Figure 2:**
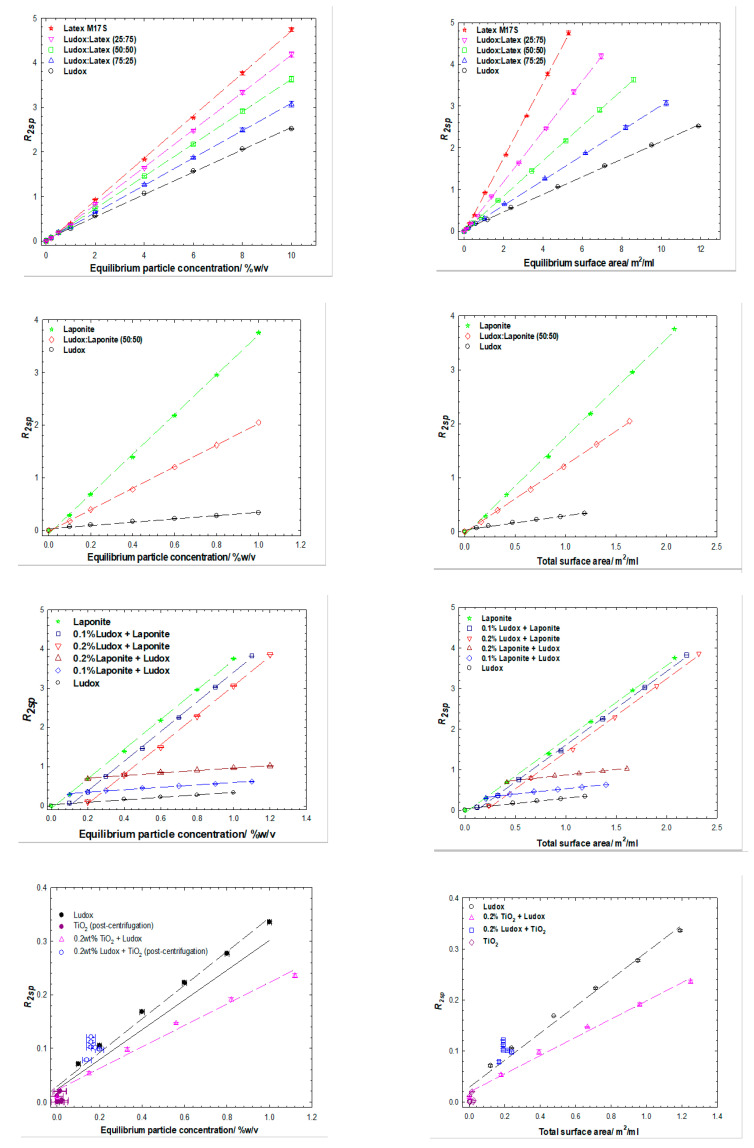
Specific relaxation rates as a function of the equilibrium particle concentration (left column) and surface area (right column) for binary particle mixtures of Ludox and latex (**top**), Ludox and laponite, (**middle** two rows) Ludox and TiO_2_ (**bottom** row). The TiO_2_ samples have been gently centrifuged to remove the TiO_2_ component.

Whilst less obvious, the same conclusion is also true for the final example—the Ludox/TiO_2_ system—where the TiO_2_ has largely been removed through centrifugation. That said, whilst the centrifugation step was designed as an opportunity to simplify the TiO_2_/silica system i.e., only one particle type would be present. In reality, this system seems somewhat more complex, due to changes in the aqueous phase (pH, ions) as a consequence of the previous presence of TiO_2_. This is illustrated with recourse to non-centrifuged samples (in which settling is evident). We will return to this point in the next section, but for context, two series of simple Ludox are included in Figure 2, one in which the dispersion has been extensively dialyzed, a second that has had the pH adjusted. The data have subtly different slopes. For the Ludox/TiO_2_ samples that have been dialyzed, both protocols return just the silica component i.e., just the varying silica component in protocol 1 (albeit with a reduced slope as alluded to above) and the constant silica component (and constant relaxation enhancement) in protocol 2. The conclusions are entirely consistent with our previous work on single heterogenous surfaces when contrasted with binary discrete homogenous surfaces [19].

### 3.2. Binary Particle Dispersions in the Presence of Polymer

**Figure 3:**
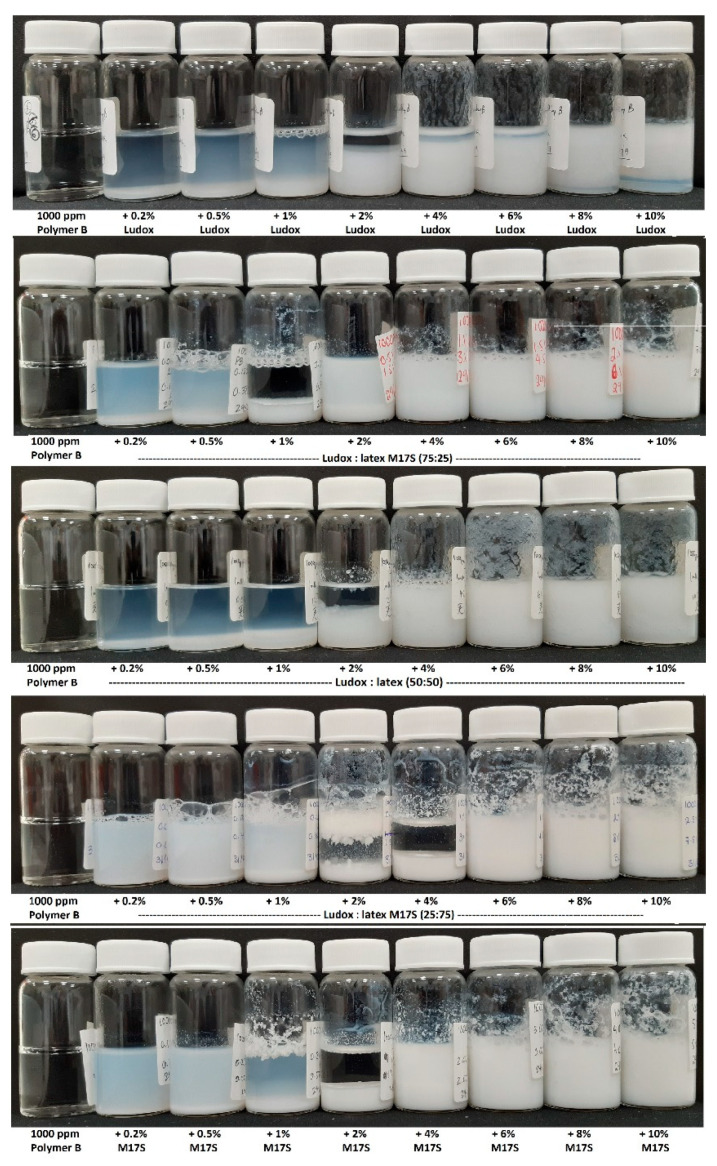
Representative phase behavior of the binary particle/polymer dispersions studied here; mixtures of 1000 ppm HEC LR (labelled “Polymer B”) with Ludox and latex as a function of increasing total particle concentration (0, 0.2, 0.5, 1, 2, 4, 6, 8 and 10% w/v (left to right) and particle ratios (Ludox:latex) (100:0, 75:25, 50:50, 25:75 and 0:100 (top to bottom).

On mixing oppositely charged polymers and particles, invariably the dispersions become unstable and some phase separation is observed. Figure 3 shows representative images from HEC LR with binary mixtures of Ludox/latex particles that illustrates the global behavior seen in these systems [9]. A distinctive visual pattern of flocculation exists—across the rows. The samples present as two coexisting phases: one particle rich, one particle devoid, except for a sample in the middle of the series that displays a disproportionately clear supernatant. On the left of these series, the cationic charge on the polymer is in excess relative to the anionic charge on the surface, whereas on the right, the surface charge is in excess. A simple interpretation of the disproportionately clear supernatant sample within the polymer concentration window is the point of charge neutralization, which reflects changes in both the total surface area present and the total surface charge. However, the visual appearance of these samples can be misleading.

A simple presentation of the data is to compare the initial and equilibrium mass fractions post-exposure to the polymer and following gentle centrifugation, Supplemental Figure S1, to quantify the amount of solid material that is rendered unstable. This analysis indicates that the polymer removes all the particles in 3 wt% Ludox and 3 wt% latex dispersions in the individual single particle systems, consistent with the similar ζ-potentials on the particles, and broadly similar amounts in their mixtures. Interestingly, the 1000 ppm polymer seems to remove less of the 75:25% dispersion and more of the 25:75% dispersion. At the lower polymer concentration of 100 ppm, a similar ratio exists *viz* 0.2 wt% Ludox. Crucially, the laponite system shows a much lower phase separation, which may be due to either a lack of adsorption or a concomitant reduction in the destabilization of the particle system, Supplemental Figure S2.

Following the previous experimental approach [9], all samples were gently centrifuged to yield a dense phase containing the flocculated species and a coexisting stable dispersion. The stable dispersion was then isolated, as either a supernatant or sub-natant. That component of the system was then characterized by both dry weights and solvent relaxation NMR and compared to the single particle comparators. The previous study [9] demonstrated that these surfaces were devoid of polymer—certainly at the higher concentrations considered. Hence, in the binary particle case, it is hypothesized that a similar dispersion with an equivalent mass fraction when compared to a comparator no-polymer case should allow an assessment of the particle types present, and, thereafter, whether preferential adsorption has occurred due to differences in the properties of the particle types (density, particle size, surface area). [The Ludox/TiO_2_ system offers an additional benefit that, due to the density of the TiO_2_, all that component can be removed during such gentle centrifugation, whether or not it bears any adsorbed polymer.]

Limiting behaviors can be anticipated in this experimental approach if one assumes no synergistic interaction between the two particles; i.e., adsorption to the two particle surfaces follows the two single particle surface cases but is modulated by the composition of the polymer/particle dispersion. At high Z, defined as cationic to anionic charge ratio, i.e., excess polymer, the majority of the particles will be removed from the solution, and the relaxation data will indicate a lack of enhancement in the relaxation time of the solvent. The dry weights analysis will indicate a very dilute particle dispersion. One would, therefore, not expect to observe any discernment in polymer behavior at these low equilibrium particle concentrations. At low Z, the polymer/particle interaction will be saturated and the dispersion will contain an appreciable mass of particles, which will then have a measurable effect on the relaxation time of the solvent. The dry weights analysis will record the presence of the particles, though not the composition. Under such sparse levels of polymer, one might expect the greatest discernment in polymer behavior to be evident, such that the less-preferred surface would be present at a higher level. By comparison with the relaxation behavior for the no-polymer “calibrants”, an analysis of the presence of any shift in the populations should be evident, though perhaps not offer a unique solution (i.e., different compositions could lead to the same overall mass fraction). 

A lack of a preference for the polymer would suggest that the *initial* and *equilibrium* particle populations would be similar, and whilst offset in total volume fraction, would be accounted for in presenting the relaxation rate determination as a function of the mass fraction. However, where the polymer shows a preference for a particular surface, one might expect the *equilibrium* population of the particles to be richer in the less favored surface, and this perturbation in the particle population to be dependent on the relative concentrations of the particle and polymer. Any polymer-induced preferential-aggregation leading to perturbations in the particle populations might be expected to be strongest in the region just above the point of electrical neutrality, Z = 1. The magnitude of the effect also depends on the sensitivity of the NMR technique for a specific surface, *via* $R_{2sp}^{enh}$, e.g., equivalent changes in *R_2_* will arise from 2 *equivalents* of silica or 1 *equivalent* of latex (measured in terms of particle concentration or surface area) given that the mass of particles removed from the dispersion by 1000 ppm polymer, varies only weakly across the particle types; 3 wt.% Ludox, 3 wt.% latex; similarly for those systems studied at lower polymer concentrations i.e., 100 ppm, Supplemental Figure S2.

### 3.3. Ludox/Latex

Following centrifugation, the equilibrium particle population from these samples might be expected to contain both Ludox and latex, due to the similarity in the mass of particles removed by the 1000 ppm polymer in the single particle cases. For each (single) average measured relaxation rate *via* comparison with Figure 2, it is evident that this single relaxation rate could pertain to a range of dispersions with differing composition and total particle concentration, i.e., a horizontal slice through those data. However, determining the equilibrium total particle concentration significantly limits those possible solutions. 

**Figure 4:**
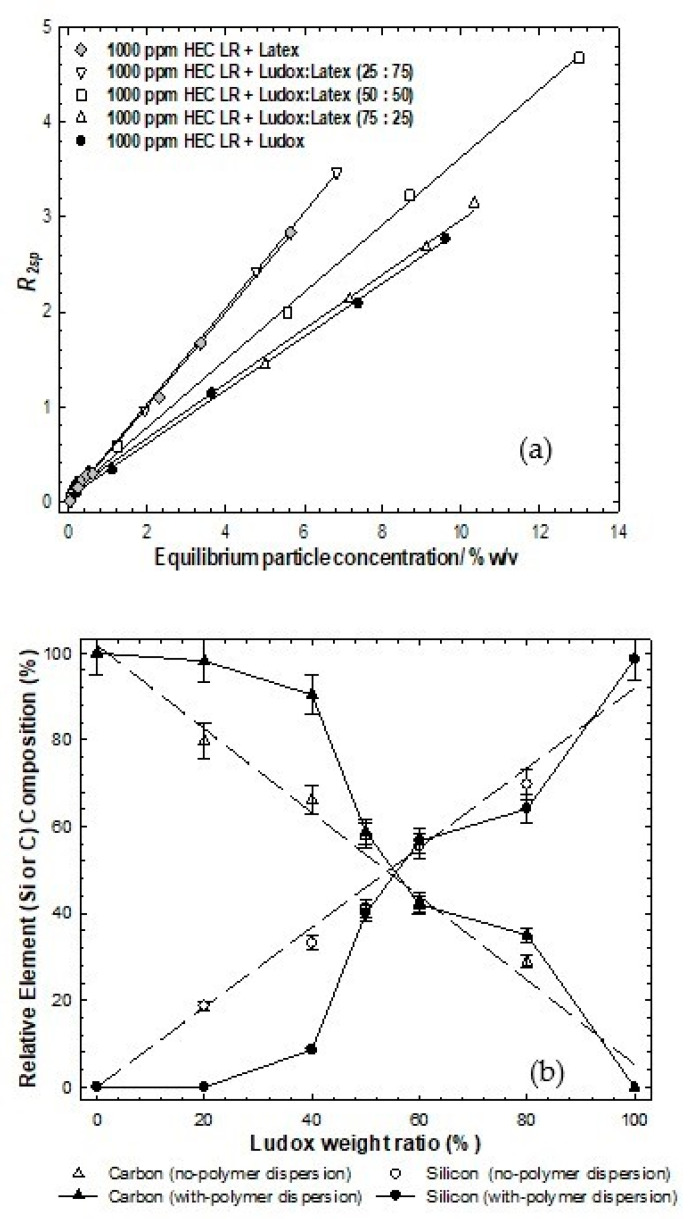
(**a**): Solvent-specific relaxation rates as a function of the equilibrium particle concentration for binary particle mixtures of Ludox post-centrifugation and exposure to 1000 ppm HEC LR. The lines are simple linear regressions to aid the eye; (**b**): EDAX data for a series of binary Ludox/latex particle mixtures with initial particle weight fraction of 10 wt% post-centrifugation and exposure to 1000 ppm HEC LR.

Figure 4a presents the specific relaxation rates as a function of the equilibrium particle concentration for the stable particle dispersions after exposure to the polymer and following centrifugation (to remove just the aggregated species). As in Figure 2 (no polymer case), the different ratios of the binary particle dispersions are located between the individual limiting particle behaviors. However, in contrast to the no-polymer case, the relaxation rate profile seems to be dominated by the more abundant particle in the mixture, i.e., the 25:75% Ludox:latex case closely resembles the simple (100%) latex case, likewise the 75:25% pairing and silica. In order words, one may conclude that the population of the excess particles in the dispersion is seemingly dominated by the more abundant particle. This would inherently also account for why the 50:50 blend lies equally between the limiting cases and is coincident with the no-polymer data. It is not possible to convert these particle concentrations into surface areas, as the compositions of the particle populations are unknown.

On the face of it, the natural interpretation of the coincidence of the relaxation data is compelling evidence that the particle population in the stable phase is predominantly the more abundant species, implying no, or, at most, a weak preference for one species. A direct and independent measure of the particle composition can be obtained by EDAX Figure 4b.

Elemental analysis of dried samples, however, present an ostensibly different picture. At 10 wt% particles/1000 ppm polymer, the lack of any silicon component in the EDAX signal for the 25:75% binary system indicates an absence of silica in the dried sample, implying that all the Ludox (2.5 wt% at a total of 10 wt%) has been preferentially removed by the 1000 ppm polymer. This would be consistent with single particle system behavior which showed that 1000 ppm polymer can accommodate up to 4 wt% particles. Therefore, the relaxation data would suggest that the equilibrium particle population would be very rich in latex, which it is.

Consider then the 75:25% binary system. The isotherm data indicate that less material is removed from the dispersion by the 1000 ppm polymer, around 2 wt%. The EDAX data show a slightly reduced signal (c60%) from the silicon, arising from a slight preference of the polymer for the latex surface. Using the same argument, the 1000 ppm polymer would then remove at the limit of preference, 2 wt% of the total 7.5 wt% silica present, leaving behind circa 5.5 wt% silica and 2.5 wt% latex. 

**Figure 5:**
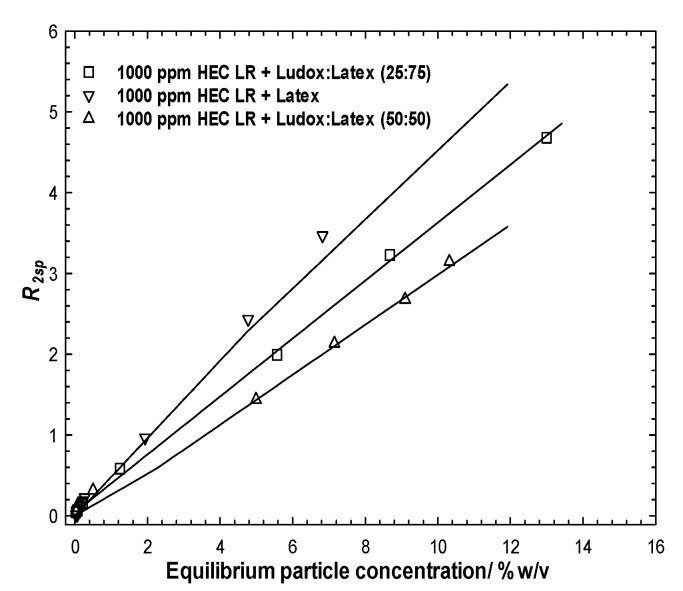
Calculated relaxation rate data for the Ludox/latex particle mixtures presented in Figure 2 i.e., initial particle weight fraction of 10 wt% post-centrifugation and exposure to 1000 ppm HEC LR assuming a 25% greater probability of adsorbing to the Ludox surface up to a degree commensurate with the single particle limit.

Figure 5 presents the results in a simple modeling of this behavior. The particle populations are calculated assuming a superposition of the two individual behaviors modulated by a 25% greater probability that the HEC LR adsorbs to the Ludox, and thereafter, the average relaxation rate is calculated from these populations using Equation (4). Whilst the electrostatic interaction is likely to dominate the interaction, and the ζ-potential is rather similar for the two materials, one could hypothesize that the slight preference for the silica surface arises due to the favorable interaction between the silanol groups and the ether oxygens on the HEC, *plus* the entropic contribution arising from the interaction of similarly sized species (the point of zero charge is approximately 1 polymer:1 particle in the Ludox case, but 50:1 in the latex case).

This simple modeling goes a long way to accounting for the gross features in the data, but there are subtle differences between the simulation and the data, especially the linearity of the simulations, notwithstanding experimental sampling and resolution factors. The non-linearity in the simulations seem most significant at lower equilibrium C*particle*, as expected, based on the foregoing discussions.

### 3.4. Ludox/Laponite

**Figure 6:**
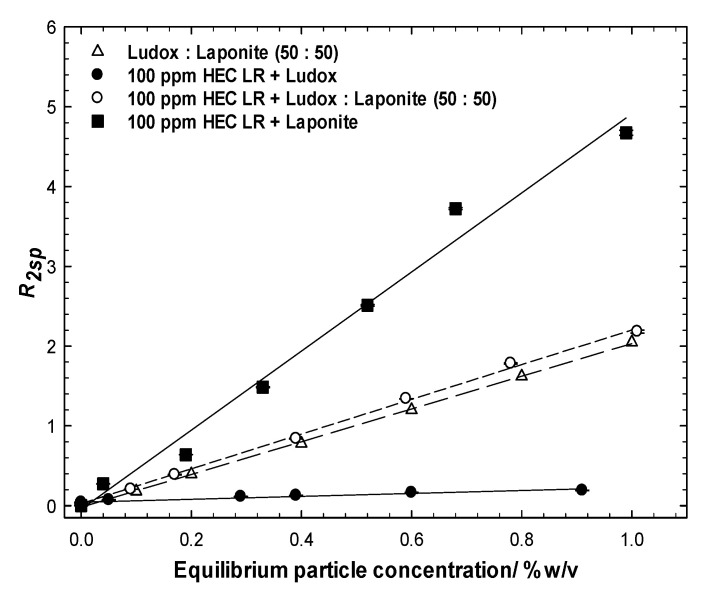
Comparison of the solvent-specific relaxation rates of Ludox:laponite (50:50) mixtures with their mixtures with the polymer. Mixtures of the polymer with the individual particles have been included for comparison.

**Figure 7:**
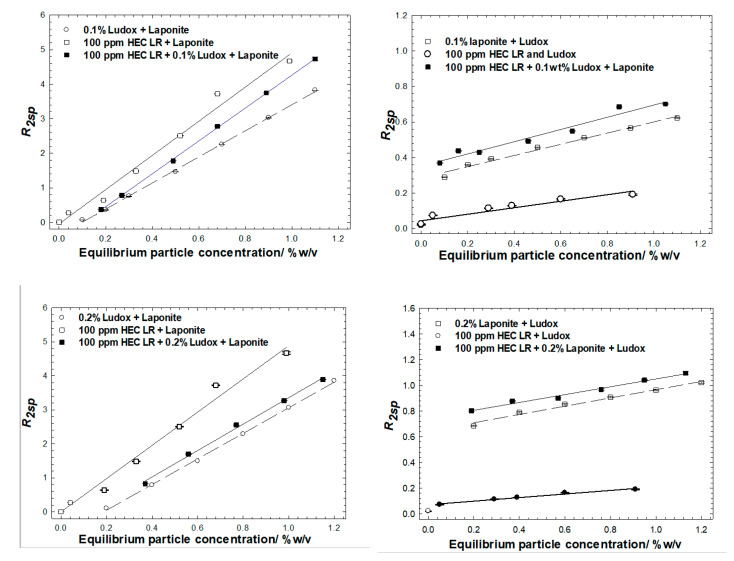
Solvent-specific relaxation rates of mixtures of HEC LR with Ludox and Laponite particles. Two series of experiments have been conducted, based on fixed concentrations of Ludox and Laponite; 0.1 wt% top row, 0.2 wt% bottom row; fixed Ludox concentration left hand column, fixed Laponite concentration, right hand column.

A matrix of HEC LR/Ludox/laponite blends, at lower C*particle*, were also studied in which the ratio of the particles was held fixed, Figure 6, or the concentration of one of the particles was fixed at either 0.1 or 0.2 wt%, Figure 7. The polymer concentration was accordingly fixed at 100 ppm in all the samples, Figure 6. These particle concentrations were chosen as they guarantee excess particles for each particle type after mixing with the polymer, and should produce a significant difference in equilibrium particle concentration after centrifugation.

The *R_2sp_* data for all the 50:50% sample mixtures are presented in Figure 6, alongside data for the comparator single- and binary-particle mixtures with/without the polymer, i.e., protocol 1. Clearly, the with-polymer and no-polymer overlap (within experimental error) indicates that exposure to the polymer and removal of some particulate material does not perturb the overall *R_2sp_ vs*. equilibrium particle concentration behaviour. Had there been a preferential removal of one particle type, the with-polymer and no-polymer behaviors would not overlay. Thus, we conclude that HEC LR exhibits no preference for the laponite or Ludox surfaces. 

Further evidence for this conclusion is seen in the data recorded from experiments conducted *via* protocol 2. In most cases, the polymer/Ludox/laponite mixtures have the same slope(s) to the equivalent Ludox/laponite dispersions, and shifted laterally and vertically when in the presence of the fixed concentration component. Consider the 0.1 wt% Ludox series, Figure 7 top left; (i) there are *two* characteristic slopes in the *three* sets of data, one for the bare particle system and one for the two with-polymer series, and (ii) the two fixed Ludox concentration datasets seem to intercept the x-axis at 0.1 wt% particles. Collectively, this suggests that the dominant interaction is between the polymer and the silica. The similar slopes in the two with-polymer cases reflects the additional surface being perceived in the NMR analysis is that being added (i.e., Laponite) and the vertical offset reflects that there is a greater prevalence of that surface that brings the greater enhancement (i.e., Laponite). Finally, the magnitude of the enhancement is reduced in the with-silica case (i.e., comparing the 100 ppm HEC LR + Laponite with the 100 ppm HEC LR + 0.1 wt% Ludox + Laponite) as a fraction of the polymer has been removed due to the adsorption/aggregation/centrifugation process onto the silica surface. Identical observations may be drawn from the 0.2 wt% Ludox series, Figure 6, bottom left, but the differences are greater due to the higher particle loadings; again *two* characteristic slopes within the *three* datasets, and now the data intercept the x-axis at 0.2 wt%.

Rather unexpectedly, the Ludox-only and Ludox-polymer cases seem to have slightly different slopes, with the polymer case being *less than* the polymer-free case. This behavior is at odds with the behavior at higher concentrations of particles (though the same polymer/particle ratio) and indicates a *loss* of surface at the *same* mass fraction. It is not immediately obvious why this would be the case, bar a change in pH or ionic strength (see Figure 2). It is not due to the presence of laponite, which would introduce the opposite switch in behavior. Possible, but less likely, reasons for such a loss of surface area could be partial surface coverage leading to incomplete phase separation (but these are polymer-rich conditions), or polymer-induced particle size fractionation (where the smaller particles are preferentially adsorbed/removed), though that would be inconsistent with the SANS data on Supplemental Figure S3. 

Notwithstanding this peculiarity, collectively, the slopes and lateral/vertical offsets evident in these data suggest that the particle population in the equilibrium phase of the polymer/binary particle mixtures, and the equivalent no-polymer binary particle dispersions, are extremely similar.

### 3.5. Ludox/TiO_2_

**Figure 8:**
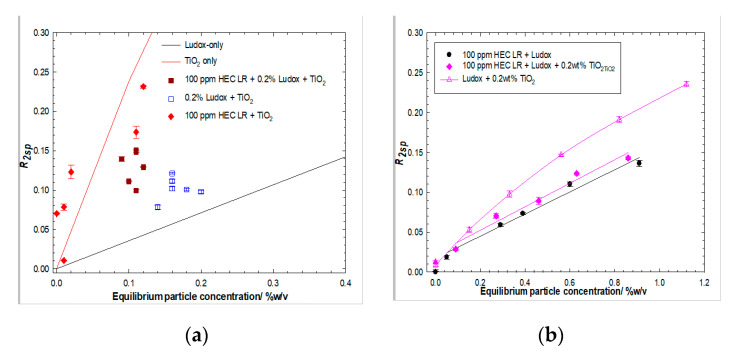
Solvent-specific relaxation rates of Ludox/TiO_2_ binary particle systems in the absence and presence of 100 ppm HEC LR post centrifugation, for fixed Ludox (left hand side, (**a**) and fixed TiO_2_ (right hand side, (**b**)) concentrations.

Consider now, the Ludox/TiO_2_ system at a comparable ratio of the various species, but at a much lower absolute C*particle* and *C_polymer_*. Figure 8a presents the specific relaxation rates for a series of dispersions with fixed concentrations of added Ludox and post-centrifugation designed to remove the majority of the TiO_2_; i.e., dispersions with an initial 0.2 wt% Ludox fixed concentration plus varying TiO_2_, post-centrifugation result in a collection of data points centered at 0.17 wt% solids, with an *R_2sp_* comparable to the value expected for a simple silica dispersion of that particle mass fraction. Similarly, dispersions with an initial 0.2 wt% Ludox fixed concentration plus varying TiO_2_, after exposure to 100 ppm HEC LR and post-centrifugation, result in a collection of data points centered at 0.10 wt% solids, but with an *R_2sp_* slightly greater than the value expected for such a silica dispersion at that particle mass fraction, indicating that the (fixed concentration of) polymer has removed a constant mass of particles. This global behavior suggests that the Ludox/polymer interaction dominates the TiO_2_/polymer one, but that the centrifugation protocol is only just sufficient to remove the TiO_2_ “fines”, indicated by the noticeable enhancement in *R_2sp_* at very low particle mass fractions in the HEC LR/TiO_2_ case (which is contrasted with a non-centrifuged particle-only dataset, the solid line).

Figure 8b presents analogous behavior-specific relaxation rates versus particle concentrations. For Ludox/TiO_2_ blends in the presence of fixed concentrations of TiO_2_, again, post-centrifugation i.e., dispersions with an initial 0.2 wt% TiO_2_ fixed concentration plus varying Ludox, post-centrifugation, result in an *R_2sp_*. The particle concentration plot is very similar to the silica-only case. The similarity of the slopes of the solvent relaxation rates *vs* equilibrium particle concentration for HEC LR/TiO_2_/Ludox and HEC LR/Ludox suggests that the particle populations are similar, i.e., their supernatant contains just Ludox, consistent with the fact that TiO_2_ has been removed due to centrifugation.

## 4. Conclusions

The ability of solvent relaxation NMR to identify preferential adsorption of a cationic hydroxyethylcellulose polymer HEC LR to specific surfaces present in a series of binary particle dispersions (Ludox, latex, laponite, TiO_2_) has been investigated. All the binary particle mixtures studied contain Ludox particles in combination with particles that exhibit a stronger relaxation enhancement than the Ludox alone. Comparisons of the solvent relaxation rates arising from the equilibrium particle phase with unknown composition were made with dispersions of known composition from which inference may be made in terms of polymer-induced particle population shifts. In the case of HEC LR, there was no significant preference for either surface in Ludox/latex or Ludox/Laponite blends, but the TiO_2_ surface was almost exclusively preferred over Ludox.

## Figures and Tables

**Table 1:** Essential characteristics of the particles used in this study.

Particle Type	Particle Diameter/nm	ζ-Potential/mV	BET Surface Area/m^2^g^−1^
Ludox TM-50	22 (+/−2)	−45 (+/−1.0)	119
Titanium (IV) oxide, anatase	18	−42	50
Laponite-RD (Laponite)		−41	206
Styrene/acrylic latex	112	−46	54

## Data Availability

Not applicable.

## Supplementary Materials

The following supporting information can be downloaded at: https://www.mdpi.com/article/10.3390/polym14091875/s1, Figure S1: Pseudo-adsorption isotherm—the mass of particle removed from a series of Ludox-latex dispersions by gentle centrifugation after exposure to HEC LR at a concentration of 1000 ppm; Figure S2: Pseudo-adsorption isotherm—the mass of particle removed from a series of dispersions comprising silica (left) and Laponite (right) by gentle centrifugation after exposure to HEC LR at a concentration of 100 ppm.; Figure S3: Small-angle neutron scattering from binary blends of Ludox/latex dispersions and a Ludox comparator to illustrate a lack of interparticle interaction or particle size fractionation.
