# Supplementary material for: Probing Selective Adsorption in Cationic-Polymer Induced Aggregation of Binary Anionic Particulate Dispersions Using Solvent Relaxation NMR
Source: Polymers (Basel). 2022 May 4;14(9):1875. doi: 10.3390/polym14091875 (PMC9104040; doi:10.3390/polym14091875)
Supplement: Supplementary file 1 [file polymers-14-01875-s001.zip › polymers-1665117-supplementary.pdf]

Probing Selective Adsorption in Cationic-Polymer Induced Aggregation of Binary Anionic Particulate Dispersions Using Solvent Relaxation NMR

Wasiu Abdullahi [1], Martin Crossman [2] and Peter Charles Griffiths [1,*]

[1] School of Science, Faculty of Engineering and Science, University of Greenwich, Chatham Maritime, Kent ME4 4TB, UK; w.o.abdullahi@greenwich.ac.uk
[2] Unilever Research, Port Sunlight, Quarry Road East, Bebington, Wirral CH63 3JW, UK; martin.crossman@unilever.com
* Correspondence: p.griffiths@gre.ac.uk; Tel.: +44-208-331-9549

Supplemental Section

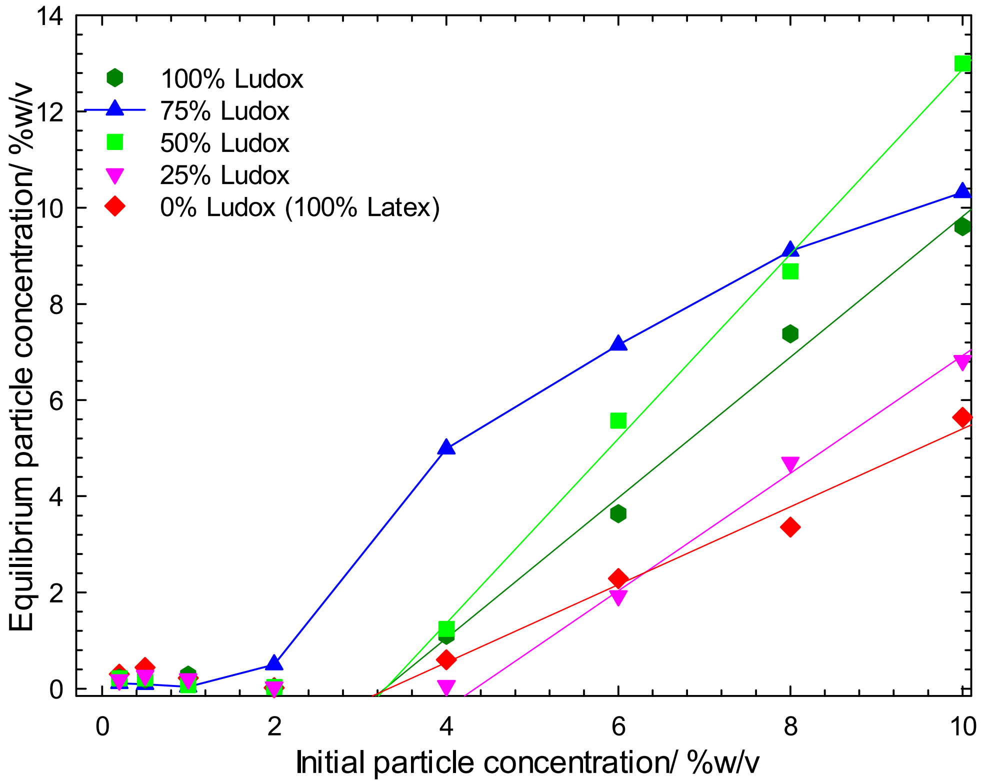

Figure S1. Pseudo-adsorption isotherm – the mass of particle removed from a series of Ludox-latex dispersions by gentle centrifugation after exposure to HEC LR at a concentration of 1000 ppm.

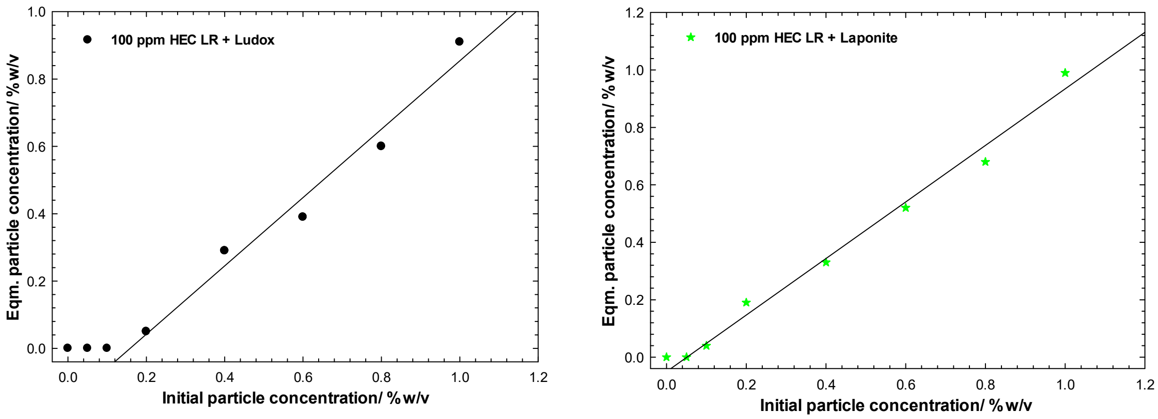

Figure S2. Pseudo-adsorption isotherm – the mass of particle removed from a series of dispersions comprising silica (left) and Laponite (right) by gentle centrifugation after exposure to HEC LR at a concentration of 100 ppm.

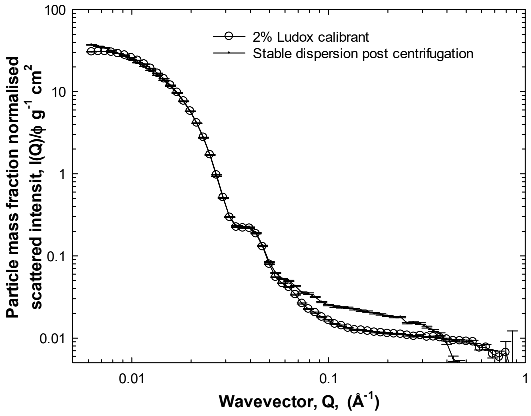

Figure S3. Small-angle neutron scattering from binary blends of Ludox/latex dispersions and a Ludox comparator to illustrate a lack of interparticle interaction or particle size fractionation.
